# Supplementary material for: Circulating MiR-30b-5p is upregulated in Cavalier King Charles Spaniels affected by early myxomatous mitral valve disease
Source: PLoS One. 2022 Jul 11;17(7):e0266208. doi: 10.1371/journal.pone.0266208 (PMC9273067; doi:10.1371/journal.pone.0266208)
Supplement: Attachment — Submitted filename: Response to Reviewers.docx [file pone.0266208.s003.docx]

**PONE-D-22-07425R1**

**Circulating miR-30b-5p is up regulated in Cavalier King Charles Spaniels affected by early myxomatous mitral valve disease**

*The authors thank the Editor and the Reviewers for their thoroughly second review of our study.*

*We are very pleased about their appreciation of the work and their positive comments about the previous review.*

*Thank you very much.*

*We have carefully considered all Reviewers’ comments and have tried to address them whenever we felt this was appropriate.*

*Best regards*

------------------------------------------------------------------------------------------------------------------------

**Review comments to the Author:**

**Reviewer #1:**

Thanks to the Authors for the work done in improving the manuscript, that can now be accepted for publication in this reviewer's opinion.

*Thank you very much for your decision, we are very pleased.*

------------------------------------------------------------------------------------------------------------------------

**Reviewer #2:** I really appreciate the changes made by the Authors and the additional information provided. Such modifications increase the quality of the manuscript and the clinical relevance of its results. I thank the Authors for their great work. However, I have some residual comments that have been listed below. The majority of them are related to the way the sentences are structured and interconnected, especially in the Abstract (which is still a little bit complex to be read and may create some confusion to readers).

*Thank you very much for your comments. We have changed the sentences as you suggested.*

Abstract

-lines 39: the word “abundance” I still present. As this term sounds a little be ambiguous, I would recommend to change it with another word, such as “amount” or a synonym.

*Thank you, we have changed this term in all the text.*

-last 3 lines of the abstract: I would suggest to modify the end of the abstract (current version “Identifying dogs with early asymptomatic myxomatous mitral valve disease through the evaluation of miR-30b-5p as new clinical research data in this breed supports more focused screening programs”) in this way (or something similar): “Identifying dogs with early asymptomatic myxomatous mitral valve disease through the evaluation of miR-30b-5p represents an intriguing possibility that certainly merits further research. Studies enrolling a larger number of dogs with preclinical stages of MMVD are needed to expand further and validate conclusively the preliminary findings from this report”.

*Thank you, we have changed this sentence as you suggested.*

Introduction

-Lines 67: I would suggest the word “interpreted” with the term “misinterpreted” (so that the sentence would become: “However, this test needs specialized equipment and well-trained operators to reduce interobserver variability, as valves affected by mild changes may be misinterpreted as normal”).

*Thank you for this suggestion. We have changed the term.*

-Lines 77-85: I would suggest to change the sentences included in these lines in this way (or something similar): “Aberrant expression of miRNAs is associated with several human [18-21] and veterinary [22-25] disorders, including cancer and heart diseases. With specific regard to canine MMVD, the dysregulation of circulating miRNAs has been previously investigated by different approaches, including real-time quantitative PCR, microarray, and next-generation sequencing [26-31]. However, it should be noticed that most of the dogs enrolled in these studies were classified as American College of Veterinary Internal Medicine (ACVIM) stages C and D, while only one previous study has investigated circulating miRNAs in adult dogs with preclinical MMVD (i.e., ACVIM stages B1 and B2).”

*Thank you for this suggestion. We have changed the sentence.*

-Line 89-93: I would suggest to change the sentences included in these lines in this way (or something similar): “It should be also highlighted that data from previous researches on dysregulation of circulating miRNAs in dogs with MMVD are partially biased by the limited sample sizes and the heterogeneity of the therapeutic protocols as well as study populations, as they were not specifically focused exclusively on one specific stage of the disease [28,29,31,33,34]”.

*Thank you very much for this suggestion. We have changed the text as you suggested.*

-Lines 93-98: I would suggest to change the sentences included in these lines in this way (or something similar): “Given the above, this study was aimed at investigating the potential use of miRNA as biomarkers to identify dogs belonging to a specific ACVIM class, namely the stage B1. The decision to focus on this ACVIM class was driven by the fact that these dogs are most subjected to breed screening, and therefore are targeted as potential breeders. Moreover, the inclusion of a single ACVIM class offered the advantage of investigating miRNA in a very homogenous study population, which, in this case, is also free from the potential confounding effects of cardiovascular drugs (as ACVIM stage B1 do not need medical treatment for their underlying structural heart disease [reference of ACVIM guidelines 2019])”.

*Thank you for this comment. We have modified this paragraph as suggested and we have added the correct reference.*

-Lines 98-104: I would suggest to deleted sentences included in these lines as the sound a little bit repetitive and not very useful in light of the topic and aim of the study (from “However, the disease goes undetected in subjects….” to “….will favour a focused screening of the subjects”).

*Thank you, we have decided to follow your suggestion to delete this part from the introduction to be less repetitive.*

-Lines 106-110: I would suggest to modified these lines in this way (or something similar): “To further standardize our study population we also selected a specific canine breed, namely the CKCS, being the most commonly affected by MMVD [pertinent references]. Lastly, we focused our analysis on three specific miRNAs previously demonstrated to be associated with canine MMVD [29,31,33] and arbitrarily divided our study population in three distinct age categories (see below for further details) to explore how these miRNAs are modulated in the plasma of CKCS of different ages affected by MMVD at stage ACVIM B1”.

*Thank you very much for this comment. We have modified this part of the introduction as you suggested.*

Discussion

-Lines 343-345: I have some perplexity about this sentence: “We demonstrated that miR-30b-5p could discriminate among ACVIM stage A CKCSs and ACVIM stage B1 CKCSs younger than 3 years, without audible heart murmurs and other clinical signs.” Specifically, my perplexity is about the “other clinical signs”. Indeed, dogs with MMVD at stage B1 do no have clinical signs at all, apart from the typical left-sided systolic heart murmur. Therefore, I would suggest deleting “and other clinical signs”.

*Thank you, we have deleted “and other clinical signs”.*

-Lines 370-371: I would suggest to modify the sentence in this way: “without evading the echocardiographic evaluation, which undoubtfully remains the gold standard for MMVD diagnosis”.

*Thank you, we have changed this sentence.*

-Line 375: correct the current version: you can write “contribute to treat” or “contribute treating”, while contribute to treating sounds wrong.

*Thank you for this suggestion. We have changed it in “contribute to treat”.*

-Line 376: replace the word “consequently” with the term “hopefully” or “theoretically”.

*Thank you, replaced with “hopefully”.*

-Lines 377-378: Modify the sentence, so that the final result would be “If confirmed, this last result would be revolutionary as it would radically change the prognostic perspectives of…”

*Thank you, we have modified the two sentences as you suggested.*

-Lines 385-386: I would further underline the fact that, currently, a widely available, rapid, reliable, not expensive kit for miRNAs analysis is lacking. Therefore, at the end of the sentence at line 386, I would write something similar to this: “Indeed, it should be note that, to date, a rapid, not expensive, widely available diagnostic kit to measure the miRNAs amount is not available in small animal practice; therefore, such an analysis is currently limited to few highly specialized laboratories”.

*Thank you for this suggestion. We completely agree with the reviewer, and we have added this sentence.*

-Lines 406-410: I would suggest to change these sentences in this way (or something similar): “Furthermore, in human medicine it has been demonstrated a potential link between patient’s age and miRNAs profile. For example, it has been showed that miRNAs may play a role in the highly coordinated mechanisms regulating genes involved in children’s development [55]. Regrettably, in veterinary medicine, to date, such a link has not been investigated. For this reason, veterinary studies purposefully designed to take into account subjects’ age categories and investigate the link between age and miRNAs profile are needed”.

*Thank you. We have modified this sentence as suggested.*

Conclusions

I believe that the conclusions of the study are overall out of topic. A study’s conclusion should primarily resume the key points relates to the main results of the study, so that readers can have some concise, clear take of message of the current research. In contrast, conclusions should not include speculations about the future and the possible, theorical application on the results of future studies which will be based on the present manuscript. Obviously, a sentence on the future prospective is desirable, but it should be a minimal part of the conclusion section, not its predominant/entire one. Therefore, I kindly invite Authors to rewrite their conclusions.

*Thank you for this comment. We have modified the conclusion section as suggested trying to be more focused on the key points of this study:*

*“The growing interest in developing new molecular markers of heart disease in young dogs affected by MMVD has led to study the expression of 3 circulating microRNAs and their application as potential biomarkers in the plasma of CKCS with early asymptomatic MMVD (ACVIM stage B1). The hypothesis that healthy dogs have different microRNA expression profiles than B1 subjects of the same breed and that the microRNA profiles can differ within the same class among subjects of different ages have been confirmed. The amount of miR-30b-5p is significantly higher in ACVIM B1 CKCS than in ACVIM A subjects, and according to the age, the amount of miR-30b-5p is higher in group B1 younger than 3 years, B1 between 3 and 7 years, and B1 older than 7 years than in group A. These results lay the basis for future studies aimed at reaching more substantial data that will help the CKCS breeders in their targeted selection programs based on the echocardiographic evaluation and to obtain healthier subjects with a reasonable life expectancy. At the same time, highlighting the risk of developing the disease at an earlier stage will favour a focused screening of the subjects. To that end, identifying early biomarkers for premature MMVD would be a helpful addition”.*

------------------------------------------------------------------------------------------------------------------------

*Thank you very much for your appreciation of the work, we are really pleased. Thank you for your precise and interesting comments.*
*We believe that the work has been greatly improved by the reviews and we thank the reviewers for their indispensable work.*
